# 人类疱疹病毒感染在造血干细胞移植后难治性肠道移植物抗宿主病中的作用及诊断和治疗

**DOI:** 10.3760/cma.j.cn121090-20240906-00339

**Published:** 2024-11

**Authors:** 海 何, 建平 张, 志杰 魏, 岳 卢, 艳丽 赵, 瑞娟 孙

**Affiliations:** 河北燕达陆道培医院骨髓移植科，廊坊 065300 Department of Bone Marrow Transplantation, Hebei Yanda Lu Daopei Hospital, Langfang 065300, China

**Keywords:** 人疱疹病毒, 肠道, 移植物抗宿主病, 异基因造血干细胞移植, Human herpesvirus infection, Gastrointestinal, Graft-versus-host-disease, Allogeneic hematopoietic stem cell transplantation

## Abstract

**目的:**

探讨人类疱疹病毒（HHV）感染在造血干细胞移植后难治性肠道移植物抗宿主病（GI-GVHD）中的作用及诊断和治疗。

**方法:**

对2022年3月至2024年7月，于河北燕达陆道培医院行异基因造血干细胞移植（allo-HSCT）治疗后出现难治性GI-GVHD并行结肠镜检查及黏膜活检的42例患者资料进行回顾性分析；采用RQ-PCR方法检测组织匀浆HHV6、HHV7、巨细胞病毒（CMV）、EB病毒（EBV）；肠黏膜进行病理检查并采用免疫组化方法检测CMV早期抗原、CMV晚期抗原，原位杂交法检测EBV。

**结果:**

42例患者纳入研究，男25例，女17例，中位年龄26（1～59）岁，均经组织病理诊断为GI-GVHD。其中34例（81.0％）合并病毒性肠炎，EBV阳性率52.4％，HHV6阳性率38.1％，CMV阳性率26.2％，HHV7阳性率14.3％。17例（40.5％）为混合病毒感染，包括5例EBV+HHV6，3例CMV+HHV6，3例CMV+EBV，2例CMV+EBV+HHV6，2例EBV+HHV6+HHV7，1例EBV+HHV7，1例HHV6+HHV7。17例（40.5％）为单一病毒感染，其中EBV感染9例、CMV感染3例、HHV6感染3例、HHV7感染2例。17例（40.5％）患者组织病理病毒检测阳性，包括7例（16.6％）CMV阳性，12例（28.5％）EBV阳性（其中2例CMV、EBV双阳性）。34例组织匀浆病毒阳性患者均可于粪便中检测到相同病毒，血液相同病毒阳性率17.6％。以组织匀浆病毒检测为肠炎诊断标准，血液检测CMV、EBV、HHV6、HHV7敏感性分别为45.4％、4.5％、6.3％、0％，特异性分别为90.3％、95.0％、100％、100％，粪便检测CMV、EBV、HHV6、HHV7敏感性和特异性均为100％。基于病因学进行治疗，腹泻的总反应率和完全缓解率分别为76.1％（32/42）和66.6％（28/42）。42例患者中位随访时间为13（1～49）个月，28例患者存活，2年预期生存率为61.9％。

**结论:**

除GVHD本身外，肠道HHV感染是GI-GVHD难治性的原因之一。血液和组织中病毒检测呈明显分离现象，即使GI-GVHD患者血液病毒检测阴性，也不能排除合并病毒性肠炎的可能。

## Abstract (en)

**Objective:**

This study aimed to investigate the role of human herpesvirus（HHV）infection in refractory intestinal graft-versus-host disease（GI-GVHD）after hematopoietic stem cell transplantation（HSCT）and its diagnosis and treatment.

**Methods:**

This study retrospectively analyzed patients presenting with refractory GI-GVHD after allogeneic HSCT（allo-HSCT）with concomitant colonoscopy and mucosal biopsy at Lu Daopei Hospital, Yanda, Hebei, from March 2022 to July 2024. Human herpesvirus 6（HHV6）, HHV7, cytomegalovirus（CMV）, and Epstein-Barr virus（EBV）detection with the RQ-PCR method. The intestinal mucosa was pathologically assessed and immunohistochemistry was utilized to detect the CMV early antigen, CMV late antigen, and EBV by in situ hybridization.

**Results:**

This study included 42 patients, consisting of 25 males and 17 females with a median age of 26（1–59）years. All were histopathologically diagnosed as GI-GVHD. Among them, 34（81.0％）cases had combined viral enteritis, with 52.4％ positive for EBV, 38.1％ positive for HHV6, 26.2％ positive for CMV, and 14.3％ positive for HHV7. Further, 17（40.5％）cases had mixed viral infections, including 5 EBV+ HHV6, 3 CMV+HHV6, 3 CMV+EBV, 2 CMV+EBV+HHV6, 2 EBV+HHV6+HHV7, 1 EBV+HHV7, and 1 HHV6 + HHV7 cases. Furthermore, 17（40.5％）had a single viral infection, including 9 EBV, 3 CMV, 3 HHV6, and 2 HHV7 cases. Moreover, 17（40.5％）patients exhibited a positive histopathological viral test, including 7（16.6％）CMV-positive and 12（28.5％）EBV-positive cases. The same positive virus was detected in the feces of all 34 patients with positive tissue homogenate virus, and the positive rate of the same virus in the blood was 17.6％. Tissue homogenized virus testing was utilized as the diagnostic criterion for enterocolitis: blood tests for CMV, EBV, HHV6, and HHV7 demonstrated a sensitivity of 45.4％, 4.5％, 6.3％, and 0％, and specificity of 90.3％, 95％, 100％, and 110％, respectively. Additionally, fecal tests for CMV, EBV, HHV6, and HHV7 demonstrated a sensitivity and specificity of 100％. Treatment based on etiology caused ORR and CR rates for diarrhea of 76.1％（32/42）and 66.6％（28/42）, respectively. The median follow-up of 42 patients was 13（1–49）months, and 28 patients survived, with an expected 2-year survival rate of 61.9％.

**Conclusion:**

In addition to GVHD itself, intestinal human herpesvirus infection is one of the reasons for the refractory nature of GI-GVHD. Viral testing in blood and tissues reveals significant segregation, and the possibility of comorbid viral enteritis cannot be excluded even if a patient with GI-GVHD tests negative for blood viruses.

异基因造血干细胞移植（allo-HSCT）是血液系统恶性肿瘤和非恶性骨髓造血衰竭疾病的一种有效治疗手段[1]。移植物抗宿主病（GVHD）是导致移植治疗失败的主要原因，尤其是难治性肠道移植物抗宿主病（GI-GVHD）。除GVHD本身外，多种因素可导致GI-GVHD难治，如病毒感染、血栓性微血管病（TMA）、移植后淋巴细胞增殖性疾病（PTLD）等，在病毒感染因素中，既往研究重点往往聚焦单一病毒[2]–[4]。本研究通过回顾性分析我院allo-HSCT后出现难治性GI-GVHD患者的临床资料，分析人类疱疹病毒（HHV）感染在难治性GI-GVHD中的作用及诊断、治疗。

## 病例与方法

一、研究对象

回顾分析2022年3月至2024年7月在河北燕达陆道培医院连续接受allo-HSCT治疗的患者资料。共纳入42例移植后出现难治性GI-GVHD的患者，所有患者均经结肠镜检查及组织病理明确诊断[5]。收集的临床资料包括患者的年龄、性别、诊断、移植类型、移植后结肠镜检查时间、GI-GVHD和分级、胃肠道活检结果、肠黏膜病毒检测结果、GI-GVHD治疗反应。

二、GVHD预防和治疗

同胞全相合移植使用短程甲氨蝶呤联合环孢素A或他克莫司预防GVHD[6]。单倍体和无关供者移植还需要加用兔抗人胸腺细胞免疫球蛋白或抗人Ｔ细胞兔免疫球蛋白、霉酚酸酯。GVHD治疗方法按照文献[7]，一线治疗为甲泼尼龙，二线治疗包括巴利昔单抗、芦可替尼、利妥昔单抗、重组人Ⅱ型肿瘤坏死因子受体抗体融合蛋白（TNFα）。

三、感染预防及治疗

于预处理开始时给予盐酸小檗碱、左氧氟沙星清除肠道宿主细菌。−10 d至−2 d开始给予更昔洛韦预防巨细胞病毒（CMV）血症。+1 d开始给予阿昔洛韦预防疱疹病毒感染。+1、+11、+21 d分别给予免疫球蛋白增强免疫力。细胞植入后给予更昔洛韦或膦甲酸钠用于CMV血症的抢先治疗，利妥昔单抗用于EB病毒（EBV）血症治疗。

四、疱疹病毒检测

1. 血液标本：移植后应用实时荧光定量PCR（RQ-PCR）检测血液中的EBV-DNA和CMV-DNA载量，试剂盒由上海之江生物技术有限公司提供。血液中EBV-DNA和CMV-DNA的阈值为500个拷贝/ml。移植后前3个月每周监测血液中的EBV-DNA和CMV-DNA载量2次，移植后第4～9个月每2周监测1次，第10～12个月每月监测1次。一旦血液中的EBV-DNA或CMV-DNA呈阳性，则每周监测2次病毒载量。

2. 粪便标本：腹泻发生后采用RQ-PCR方法检测HSV-1、HSV-2、VZV、EBV、CMV、HHV6～8在粪便中的核酸，每周1次。粪便中病毒载量的阈值与在血液中使用的阈值相同。

3. 组织匀浆标本：肠黏膜组织送病理科进行病理检查，同时采用RQ-PCR检测肠道组织匀浆中HSV-1、HSV-2、VZV、EBV、CMV、HHV6～8。组织匀浆中病毒载量的阈值定义参照血液中的阈值。为排除血液中病毒影响，如果血液检测为阴性，则组织匀浆中病毒载量的阈值与血液中的阈值相同；而如果血液病毒检测为阳性，则组织匀浆中病毒载量高于血液中被定义为阳性。

五、结肠镜检查及肠黏膜组织活检

所有患者均进行结肠镜检查，活检组织取自结肠的解剖节段，特别是剥脱病变或溃疡基底黏膜部位[7]。肠黏膜组织送病理科病理检查，应用免疫组化检查CMV，原位杂交技术检查EBER。同时采用RQ-PCR检测肠道组织匀浆中HSV-1、HSV-2、VZV、EBV、CMV、HHV6～8。

六、评价及定义和治疗

肠道GVHD的临床和组织病理学分级根据指南标准[5],[8]定义。病毒性肠炎的诊断依据文献[9]–[11]标准定义。疱疹病毒治疗依据文献[11]。根据指南[7]对GI-GVHD的治疗反应进行评估，包括完全缓解（CR）、部分缓解（PR）和无反应（NR）。CR定义为每24 h少于2次稠糊大便或者成形便。PR定义为每天的排便频率低于治疗前的频率和（或）腹泻量对比治疗前减少25％以上，但未达到CR。NR定义为每天排便频率、每天腹泻量与治疗前相似或更差。总反应率（ORR）为CR率+PR率。总生存（OS）率定义为造血干细胞回输结束至末次随访或死亡的时间。难治性GI-GVHD的定义：①GVHD经糖皮质激素治疗3 d进展或治疗7 d后无改善；②GVHD治疗期间腹泻暂时改善，但随后恶化；③GVHD经二线免疫抑制药物治疗7 d后无改善或进展。病毒阳性定义为血液中EBV-DNA和CMV-DNA>500个拷贝/ml。

七、随访

采用电话或查阅病历的方式进行随访，随访截止时间为2024年7月30日。

八、统计学处理

采用SPSS 22.0软件进行统计分析。定量变量用中位数（范围）表示，定性变量用百分比表示。采用Kaplan-Meier法计算生存率。

## 结果

1.患者临床特点：42例患者进行了45例次内镜检查，其中3例患者接受了2次内镜检查。男25例，女17例，中位年龄26（1～59）岁。原发病包括再生障碍性贫血4例，急性淋巴细胞白血病12例，急性髓系白血病19例，慢性粒-单核细胞白血病2例，骨髓增生异常综合征3例，急性混合细胞白血病2例。移植类型包括同胞相合供者移植6例，无关供者移植6例，亲缘单倍体移植32例。难治性GI-GVHD的中位发病时间为移植后52（15～301）d。19例患者出现单纯肠道GVHD，12例患者出现皮肤、肠道GVHD，5例患者出现肝脏、肠道GVHD，6例患者出现皮肤、肝脏、肠道GVHD。结肠镜检查中位时间为腹泻后22（2～86）d。结肠镜检查时，12例患者未应用二线药物治疗，仅为糖皮质激素治疗无效。30例患者已应用二线免疫抑制药物，其中，接受芦可替尼治疗2例，接受巴利昔单抗治疗17例，接受巴利昔单抗、TNFα治疗4例，接受巴利昔单抗、芦可替尼治疗5例，接受巴利昔单抗、芦可替尼、TNFα治疗2例。根据腹泻临床表现，诊断为GI-GVHD Ⅱ级17例，Ⅲ级15例，Ⅳ级10例。根据组织病理表现，诊断为GI-GVHD Ⅰ级6例，Ⅱ级11例，Ⅲ级15例，Ⅳ级10例。

2.病毒检测及特异性和敏感性：组织病理检测显示，17例（40.5％）患者病毒检测阳性。CMV免疫组化7例（16.7％，7/42）阳性，EBER 12例（28.6％，12/42）阳性（其中CMV和EBER双阳性2例）。

RQ-PCR检测血液中病毒显示，GVHD期间共12例（28.6％）患者发生CMV血症，2例（4.8％）患者发生EBV血症。其中，结肠镜检查前2周内8例（19％）患者发生CMV血症，2例（4.8％）患者发生EBV血症，内镜检查前24 h经治疗均转阴。结肠镜检查后4例（9.5％）患者出现了新诊断的CMV血症。

RQ-PCR检测肠黏膜组织匀浆病毒显示，34例（81.0％）患者病毒检测阳性，11例（26.2％）CMV阳性，22例（52.4％）EBV阳性，16例（38.1％）HHV6阳性，6例（14.3％）HHV7阳性。其中，13例（31％）患者出现两种病毒混合感染，4例（9.5％）患者出现3种病毒混合感染（表1）。

**表1:** 不同标本人类疱疹病毒检测阳性情况［例（％）］

HHV	组织匀浆PCR阳性	病理病毒阳性	粪便PCR阳性	血液PCR阳性
EBV	17（40.5）	10（23.8）	17（40.5）	2（4.8）
EBV	9（21.4）		9（21.4）	2（4.8）
EBV+HHV6	5（11.9）		5（11.9）	0（0）
EBV+HHV7	1（2.4）		1（2.4）	0（0）
EBV+HHV6+HHV7	2（4.8）		2（4.8）	0（0）
CMV	6（14.3）	5（11.9）	6（14.3）	8（19.0）
CMV	3（7.1）		3（7.1）	8（19.0）
CMV+HHV6	3（7.1）		3（7.1）	0（0）
CMV+EBV	5（11.9）	2（4.7）	5（11.9）	0（0）
CMV+EBV	3（7.1）		3（7.1）	0（0）
CMV+EBV+HHV6	2（4.8）		2（4.8）	0（0）
HHV6	3（7.1）		3（7.1）	1（2.4）
HHV7	2（4.8）		2（4.8）	0（0）
HHV6+HHV7	1（2.4）		1（2.4）	0（0）

合计	34（81.0）	17（40.5）	34（81.0）	11（26.2）

**注** EBV：EB病毒；CMV：巨细胞病毒；HHV：人类疱疹病毒；肠黏膜组织病理仅检测CMV、EBV

获取组织匀浆阳性病毒结果后，再次分别进行血液、粪便样本相同病毒RQ-PCR定量。结果显示，除1例儿童患者发现HHV6血症，其余患者均无相同病毒血症发现，但粪便样本中均可检测到与组织匀浆中相同病毒。

以组织匀浆病毒检测为肠炎诊断标准，分析血液、粪便检测对病毒性肠炎诊断敏感性和特异性。在11例CMV肠炎患者中，5例结肠镜检查前曾出现CMV血症，11例粪便CMV阳性，两者的敏感性分别为45.4％、100％。在31例无CMV肠炎患者中，3例结肠镜检查前出现CMV血症，粪便均未发现CMV阳性，两者的特异性分别为90.3％、100％。在22例EBV肠炎患者中，1例结肠镜检查前出现EBV血症，22例患者粪便EBV阳性，两者的敏感性分别为4.5％、100％。在20例未发生EBV肠炎患者中，1例结肠镜检查前出现EBV血症，粪便均未发现EBV阳性，两者的特异性分别为95.0％、100％。在16例HHV6肠炎患者中，1例患者出现HHV6血症，16例患者粪便HHV6阳性，两者的敏感性分别为6.3％、100％。在26例未发生HHV6肠炎患者中，血液及粪便均未发现HHV6阳性，两者的特异性均为100％。在6例HHV7肠炎患者中，无患者出现HHV7血症，6例患者粪便HHV7阳性，两者的敏感性分别为0％、100％。在36例未发生HHV7肠炎患者中，血液及粪便均未发现HHV7阳性，两者的特异性均为100％（表2）。

**表2:** 以组织匀浆病毒检测为诊断标准下血液和粪便检测对病毒性肠炎诊断敏感性和特异性比较（％）

HHV	标本类型	敏感性	特异性	阳性预测值	阴性预测值
CMV	粪便	100	100	100	100
	血液	45.5	90.3	62.5	82.4
EBV	粪便	100	100	100	100
	血液	4.5	95.0	50.0	47.5
HHV6	粪便	100	100	100	100
	血液	6.3	100	100	63.4
HHV7	粪便	100	100	100	100
	血液	0	100	0	85.7

**注** CMV：巨细胞病毒；EBV：EB病毒；HHV：人类疱疹病毒

3.治疗转归及生存：所有患者均按病因学进行治疗，腹泻的ORR和CR率分别为76.2％（32/42）和66.7％（28/42）。单纯GI-GVHD患者5例，3例患者CR，2例死亡，1例死于消化道出血，1例死于消化道出血合并TMA。GI-GVHD合并TMA患者3例，1例患者CR，2例患者死亡，均死于消化道出血。GI-GVHD合并疱疹病毒感染患者34例，腹泻ORR和CR率分别为82.4％（28/34）和70.6％（24/34），死亡患者10例，1例死于肠梗阻，2例死于继发感染，7例死于消化道出血（表3）。42例患者中位随访时间为13（1～49）个月，28例患者存活，14例死亡，2年预期生存率为61.9％。

**表3:** GI-GVHD患者的病因学治疗结果（例）

病因	例数	总反应	完全缓解	死亡（死因）
GI-GVHD	5	3	3	2（1例消化道出血；1例消化道出血合并TMA）
GI-GVHD合并TMA	3	1	1	2（2例消化道出血）
GI-GVHD合并病毒感染	34	28	24	10
CMV	3	2	1	2（1例消化道出血；1例继发感染）
EBV	9	9	8	1（消化道出血）
HHV6	3	3	3	
HHV7	2	1	1	1（消化道出血）
CMV+HHV6	3	3	2	1（消化道出血）
CMV+EBV	3	2	2	1（消化道出血）
EBV+HHV6	5	4	4	1（消化道出血）
EBV+HHV7	1	1	1	
HHV6+HHV7	1			1（消化道出血）
EBV+HHV6+HHV7	2	1	1	1（肠梗阻）
CMV+EBV+HHV6	2	2	1	1（继发感染）

**注** GI-GVHD：肠道移植物抗宿主病；TMA：血栓性微血管病；EBV：EB病毒；CMV：巨细胞病毒；HHV：人类疱疹病毒

## 讨论

在难治性GI-GVHD患者中，GVHD难治通常归因于对免疫抑制药物的耐药，发生率约为50％，而由其他病因引起的关注较少[5],[12]–[13]。GI-GVHD和病毒感染都是allo-HSCT受者腹泻的常见原因[4],[5],[14]。它们的症状和体征重叠，在没有组织病理学检查情况下，诊断和治疗的决策很困难，特别是当GI-GVHD合并病毒感染时。本研究中纳入的患者均经肠黏膜组织病理明确诊断为GI-GVHD，发现除GVHD外，HHV感染的发生率高达81.0％，HHV感染是导致GI-GVHD难治性的原因之一。

HHV感染是HSCT后常见并发症，有研究报道，其不仅可诱发GVHD发生，还可加重GVHD难治性[4],[15]。其中，CMV是最常见的病原体，HSCT后CMV肠炎发生率为12.3％～25.2％[4],[16]。在本研究中，确诊CMV肠炎患者11例（26.2％），5例（45.4％）患者确诊前曾出现CMV血症，和上述报道类似。特别重要的是，我们发现血液和组织病毒检查呈分离现象，在未发生CMV血症组中，6例（17.6％）患者确诊CMV肠炎，在未发生EBV血症患者中，21例（52.5％）确诊EBV肠炎。这种现象表明，即使难治性GI-GVHD患者血液病毒检测阴性，也不能排除合并病毒性肠炎的可能，进一步的肠黏膜活检及组织匀浆病毒检测非常重要，且对临床治疗决策有指导意义[11],[16]–[17]。

allo-HSCT后累及肠道的EBV相关PTLD很常见，但EBV肠炎很少报道[2],[11]。令人惊讶的是，我们发现在本研究中，22例（52.3％）患者确诊为EBV肠炎。Goloshchapov等[18]报道，在119例allo-HSCT后出现腹泻患者中，35％的患者确诊为EBV肠炎，与我们的研究结果相似。EBV肠炎的高检出率可能与我们研究的群体为难治性GI-GVHD患者及应用RQ-PCR作为检测手段有关。

目前关于HHV6与GI-GVHD关系的文献报道很少。韩婷婷等[19]报道，45例allo-HSCT后出现GI-GVHD患者中，肠黏膜HHV6阳性率46.7％，但与HHV6阴性组比较，给予抗病毒治疗并未发现临床症状显著改善。而其他研究得出了相反的结论，认为HHV6与aGVHD患者较差的预后相关，需积极治疗[20]–[21]。本研究中肠黏膜HHV6阳性率38.1％，但大部分伴随CMV、EBV、HHV7感染，单一感染率仅为7.1％（3/42），3例患者经减撤免疫抑制药物、抗病毒治疗后获得腹泻缓解。HHV7感染在allo-HSCT受者中并不常见，关于HHV7疾病的报道也很少。本研究中患者肠黏膜HHV7阳性率14.3％，仅2例患者单纯HHV7感染。因此，HHV6、HHV7导致GI-GVHD患者难治需进一步研究。

目前病毒性肠炎的诊断主要依靠肠黏膜组织活检和黏膜匀浆组织PCR定量[9]–[11]，然而，移植后出现GI-GVHD患者往往身体状态差、凝血功能异常、血小板低下，肠道活检的内镜取样无法获得。研究发现，在活动性病毒感染期间，疱疹病毒脱落可发生在受感染者的尿液、呼吸道分泌物和粪便中[22]–[23]。因此，在粪便样本中检测疱疹病毒DNA协助诊断病毒性肠炎可作为一种非侵入性的诊断替代方法。我们对所有肠黏膜组织匀浆病毒阳性患者的粪便标本，进行了相同的病毒PCR定量检测，发现所有患者均可从粪便样本中检测到相同的病毒株，与其他研究结果报道一致。

GI-GVHD与病毒性肠炎并存时，治疗策略上存在很大的矛盾，前者需要加强免疫抑制治疗，而后者则需要减低免疫抑制剂用量、增强抗病毒治疗。而以往的研究往往只关注难治性GI-GVHD的二线治疗，有效率为30％～61.1％[12],[17],[24]。但我们根据难治性GI-GVHD是否合并HHV感染制定治疗策略，有效率为76.2％，疗效更优。

综上所述，我们的研究结果表明，除GVHD本身外，肠道HHV感染是GI-GVHD难治性的原因之一。血液和组织中病毒检测呈明显分离现象，即使GI-GVHD患者血液病毒检测阴性，也不能排除合并病毒性肠炎的可能，应积极进行结肠镜检查。在获得肠黏膜组织困难情况下，粪便样本中检测疱疹病毒DNA协助诊断病毒性肠炎可作为一种非侵入性的诊断替代方法。本文为回顾性研究，样本量小，需扩大样本量进一步验证。
